# Statistical and Machine Learning forecasting methods: Concerns and ways forward

**DOI:** 10.1371/journal.pone.0194889

**Published:** 2018-03-27

**Authors:** Spyros Makridakis, Evangelos Spiliotis, Vassilios Assimakopoulos

**Affiliations:** 1 Institute For the Future (IFF), University of Nicosia, Nicosia, Cyprus; 2 Forecasting and Strategy Unit, School of Electrical and Computer Engineering, National Technical University of Athens, Zografou, Greece; Universidad Veracruzana, MEXICO

## Abstract

Machine Learning (ML) methods have been proposed in the academic literature as alternatives to statistical ones for time series forecasting. Yet, scant evidence is available about their relative performance in terms of accuracy and computational requirements. The purpose of this paper is to evaluate such performance across multiple forecasting horizons using a large subset of 1045 monthly time series used in the M3 Competition. After comparing the post-sample accuracy of popular ML methods with that of eight traditional statistical ones, we found that the former are dominated across both accuracy measures used and for all forecasting horizons examined. Moreover, we observed that their computational requirements are considerably greater than those of statistical methods. The paper discusses the results, explains why the accuracy of ML models is below that of statistical ones and proposes some possible ways forward. The empirical results found in our research stress the need for objective and unbiased ways to test the performance of forecasting methods that can be achieved through sizable and open competitions allowing meaningful comparisons and definite conclusions.

## 1 Introduction

Artificial Intelligence (AI) has gained considerable prominence over the last decade fueled by a number of high profile applications in Autonomous Vehicles (AV), intelligent robots, image and speech recognition, automatic translations, medical and law usage as well as beating champions in games like chess, Jeopardy, GO and poker [1]. The successes of AI are based on the utilization of algorithms capable of learning by trial and error and improving their performance over time, not just by step-by-step coding instructions based on logic, if-then rules and decision trees, which is the sphere of traditional programming.

In light of the above, AI found applications in the field of forecasting and a considerable amount of research has been conducted on how a special class of it, utilizing Machine Learning methods (ML) and especially Neural Networks (NNs), can be exploited to improve time series predictions. Literally hundreds of papers propose new ML algorithms, suggesting methodological advances and accuracy improvements [2–8]. Yet, limited objective evidence is available regarding their relative performance as a standard forecasting tool [9–12]. Their superiority claims are characterized by the following three major limitations:

- Their conclusions are based on a few, or even a single time series, raising questions about the statistical significance of the results and their generalization.
- The methods are evaluated for short-term forecasting horizons, often one-step-ahead, not considering medium and long-term ones.
- No benchmarks are used to compare the accuracy of ML methods versus alternative ones.

The objective of ML methods is the same as that of statistical ones. They both aim at improving forecasting accuracy by minimizing some loss function, typically the sum of squared errors. Their difference lies in how such a minimization is done with ML methods utilizing non-linear algorithms to do so while statistical ones linear processes. ML methods are computationally more demanding than statistical ones, requiring greater dependence on computer science to be implemented, placing them at the intersection of statistics and computer science.

The importance of objectively evaluating the relative performance of the ML methods in forecasting is obvious but has not been achieved so far raising questions about their practical value to improve forecasting accuracy and advance the field of forecasting. Simply being new, or based on AI, is not enough to persuade users of their practical advantages over alternative methods. A similar situation has been reported by [13] for data mining methods, suggesting among others that novel approaches should be properly tested through a wide range of diverse datasets and comparisons with benchmarks. As mentioned by [14], it should become clear that ML methods are not a panacea that would automatically improve forecasting accuracy. “Their capabilities can easily generate implausible solutions, leading to exaggerated claims of their potentials” and must be carefully investigated before any claims can be accepted.

This paper consists of three sections. The first briefly reviews published empirical studies and investigates the performance of ML methods in comparison to statistical ones, also deliberating some major issues related to forecasting accuracy. The second part uses a subset of 1045 monthly series (the same ones used by [15]) from the 3003 of the M3 Competition [16] to calculate the performance of eight traditional statistical methods and eight popular ML ones, the same as those used by [15], plus two more that have become popular during recent years [17]. The forecasting model was developed using the first *n* − 18 observations, where *n* is the length of the series. Then, 18 forecasts were produced and their accuracy was evaluated compared to the actual values not used in developing the forecasting model. In addition, the computational complexity of the methods used was recorded as well as the accuracy of fitting the model to the *n* − 18 historical data (Model Fit). The third section discusses the outcome of the comparisons and attempts to explain why the forecasting accuracy of ML models was lower than most statistical ones, while also proposing possible ways to improve it. A critical question being asked is whether ML methods can actually be made to “learn” more efficiently using more information about the future and its unknown errors, rather than past ones.

The motivation for writing this paper was an article [18] published in Neural Networks in June 2017. The aim of the article was to improve the forecasting accuracy of stock price fluctuations and claimed that “the empirical results show that the proposed model indeed display a good performance in forecasting stock market fluctuations”. In our view, the results seemed extremely accurate for stock market series that are essentially close to random walks so we wanted to replicate the results of the article and emailed the corresponding author asking for information to be able to do so. We got no answer and we, therefore, emailed the Editor-in-Chief of the Journal asking for his help. He suggested contacting the other author to get the required information. We consequently, emailed this author but we never got a reply. Not being able to replicate the result of [18] and not finding research studies comparing ML methods with alternative ones we decided to start the research leading to this paper.

## 2 The accuracy of ML methods: A brief review and discussion

The first application of NNs (as ML methods were called at that time and also sometimes today) in forecasting dates back to 1964 but did not achieve much follow-up until the technique of backpropagation was introduced almost 20 years later [19]. Since then, there have been numerous studies utilizing NNs and some of them comparing their accuracy to traditional, statistical ones. A good number of these studies, going back to 1995, are summarized in the work of [15] who concluded: “The outcome of all of these studies has been somewhat mixed”. A similar conclusion was reached by [9] who evaluated 48 NN studies and stated that their accuracy in comparison to statistical methods provided mixed results. What characterized all these studies, however, was the limited number of series employed in the comparisons.

The first large scale study, using 3003 time series, dates back to the M3 Competition published in 2000 by [16] that included an Automated Artificial NN (AANN) method which, accuracy-wise, did average in comparison to the traditional statistical ones included in the Competition and below the most accurate ones (see Table 1). Eleven years later, Crone, Hibon and Nikolopoulos (C-H-N) published the results of a specialized NN competition, using a subset of the M3 monthly data [12]. In this competition they compared 22 NN and CI (Computational Intelligence) methods, in addition to 11 statistical ones. Their conclusion was that no ML method outperformed the Theta method [20], the most accurate one in the M3 Competition, and that only one [21] was more accurate than Damped trend exponential smoothing [22] when the symmetric Mean Absolute Percentage Error (sMAPE) for the average of all 18 forecasting horizons was used. However, four NNs did better than the AANN of the M3 Competition denoting improvements in the accuracy of newer ML methods. Overall, however, the accuracy of the NNs was not exceptional, vis-à-vis those of the M3 Competition, or the 11 statistical methods that were included in the (C-H-N) study (see Table 2).

**Table 1:** sMAPE across the 3003 time series of the M3 competition.

Method	Forecasting horizon										Average of forecasting horizons					
	1	2	3	4	5	6	8	12	15	18	1–4	1–6	1–8	1–12	1–15	1–18
Theta	8.4	9.6	11.3	12.5	13.2	14.0	12.0	13.2	16.2	18.2	10.4	11.5	11.6	12.0	12.4	13.0
Damped	8.8	10.0	12.0	13.5	13.7	14.3	12.5	13.9	17.5	18.9	11.1	12.0	12.1	12.4	13.0	13.6
Box-Jenkins	9.2	10.4	12.2	13.9	14.0	14.8	13.0	14.1	17.8	19.3	11.4	12.4	12.5	12.8	13.4	14.0
**AANN**	**9.0**	**10.4**	**11.8**	**13.8**	**13.8**	**15.5**	**13.4**	**14.6**	**17.3**	**19.6**	**11.2**	**12.4**	**12.6**	**13.0**	**13.5**	**14.1**
Single	9.5	10.6	12.7	14.1	14.3	15.0	13.3	14.5	18.3	19.4	11.7	12.7	12.8	13.1	13.7	14.4
Holt	9.0	10.4	12.8	14.5	15.1	15.8	13.9	14.8	18.8	20.2	11.7	12.9	13.1	13.4	14.0	14.6
Naive 2	10.5	11.3	13.6	15.1	15.1	15.9	14.5	16.0	19.3	20.7	12.6	13.6	13.8	14.2	14.8	15.5

NNs are defined with bold numbers.

**Table 2:** sMAPE and ranks of errors on the complete dataset of the C-H-N study.

Method	Average errors				Rank across all methods			
	sMAPE(%)	MdRAE	MASE	AR	sMAPE(%)	MdRAE	MASE	AR
Theta	14.89	0.88	1.13	17.8	2	3	1	2
**Illies**	**15.18**	**0.84**	**1.25**	**18.4**	**3**	**2**	**11**	**4**
ForecastPro	15.44	0.89	1.17	18.2	4	4	3	3
DES	15.90	0.94	1.17	18.9	5	14	3	6
Comb S-H-D	15.93	0.09	1.21	18.8	6	5	7	5
Autobox	15.95	0.93	1.18	19.2	7	11	5	7
**Flores**	**16.31**	**0.93**	**1.20**	**19.3**	**8**	**11**	**6**	**8**
SES	16.42	0.96	1.21	19.6	9	16	7	12
**Chen**	**16.55**	**0.94**	**1.34**	**19.5**	**11**	**14**	**18**	**9**
**D’yakonov**	**16.57**	**0.91**	**1.26**	**20.0**	**12**	**7**	**12**	**15**
**AANN**	**16.81**	**0.91**	**1.21**	**19.5**	**13**	**7**	**7**	**9**
**Kamel**	**16.92**	**0.90**	**1.28**	**19.6**	**14**	**5**	**13**	**12**

NNs are defined with bold numbers.

ML methods have been gaining prominence over time as interest in AI has been rising. They are used to predict financial series [18, 23], the direction of the stock market [24], macroeconomic variables [25], accounting balance sheet information [26] and a good number of other applications, covering a wide range of areas [27]. A major purpose of this study is to determine, empirically, if their performance exceeds that of statistical methods and how their advantages could be exploited to improve forecasting accuracy. What seems certain is that Chatfield’s prediction of NNs becoming a “breakthrough or passing fad” will not be realized [10]. Their performance cannot be classified yet as a breakthrough but at the same time they are still used while there are indications that such usage will increase over time as newer ML methods are introduced and more ways are being devised to improve their accuracy [15, 28] and computational efficiency.

## 3 The major contribution of this paper: Comparing the performance of ML forecasting methods with traditional statistical ones

As highlighted in the previous section, [15] compared in their study the performance of eight families of the ML model regarding their accuracy:

1. Multi-Layer Perceptron (MLP)
2. Bayesian Neural Network (BNN)
3. Radial Basis Functions (RBF)
4. Generalized Regression Neural Networks (GRNN), also called kernel regression
5. K-Nearest Neighbor regression (KNN)
6. CART regression trees (CART)
7. Support Vector Regression (SVR), and
8. Gaussian Processes (GP)

(for more information regarding these models see the work of [15, 29, 30] as well as our own descriptions in section 3.3 below).

In their paper, Ahmed and co-authors used a subset of 1045 series (the same ones being used in our study), selected from the monthly ones of the M3 Competition so that they have a length of between 81 and 126 months. However, before computing the 18 forecasts, they preprocessed the series in order to achieve stationarity in their mean and variance. This was done using the log transformation, then deseasonalization and finally scaling, while first differences were also considered for removing the component of trend. Consequently, they calculated one-step-ahead forecasts for each one of the 1045 series. The sMAPE and ranking of the eight ML methods can be seen in Table 3 (for details of how the preprocessing was done, how the forecasts were produced and how the accuracy measures were computed, see the paper by [15]). As seen, the most accurate ML method is the MLP, the next one is the BNN and the third the GP. The sMAPE of the remaining methods is at a double digit indicating a distinct difference in their accuracy. What would be of considerable research value is to investigate the reasons for the differences in accuracy among the eight ML methods and come up with guidelines of selecting the most appropriate one for new types of forecasting applications.

**Table 3:** Forecasting performance (sMAPE) of the ML methods tested in the study of Ahmed et.al.

Rank	Method	sMAPE(%)
1	MLP	8.34
2	BNN	8.58
3	GP	9.62
4	GRNN	10.33
5	KNN	10.34
6	SVR	10.40
7	CART	11.72
8	RBF	15.79

The major contribution of this paper is to extend Ahmed and co-authors’ study in six directions: First, we included eight statistical methods in order to compare the accuracy of the ML methods with that of the statistical ones. Second, we included two additional ML methods that have become popular during the last years [17]. These methods are the Recurrent Neural Network (RNN) and the Long Short Term Memory network (LSTM). Third, we used an additional accuracy measure introduced in recently, the MASE [31], to ensure that the same conclusions apply for such an alternative. Fourth, we used three different approaches for obtaining 18-step-ahead forecasts, not just the one-step-ahead ones computed by Ahmed and co-authors, to test the forecasting performance of ML methods for longer horizons. Fifth, we recorded a measure of computational complexity to determine the amount of computer time required by each method to develop the model and obtain the forecasts. Finally, we estimated a Model Fit measure to determine how well each method was fitted to the *n* − 18 historical data that was used for training and parameter estimation. The purpose of such a measure was to determine the relationship between the accuracy of model fitting and that of the post sample forecasting accuracy in order to figure out possible over-fitting that may decrease the accuracy of the post-sample predictions.

### 3.1 Accuracy measures

Two accuracy measures are used in this paper: The symmetric Mean Absolute Percentage Error (sMAPE) and the Mean Absolute Scaled Error (MASE). The first measure, which was originally used in the M3 Competition for evaluating the participating methods, is defined as follows:

$$sMAPE=\frac{2}{k}\sum_{t=1}^{k}\frac{\left|Y_{t}-{\hat{Y}}_{t}\right|}{\left|Y_{t}\right|+\left|{\hat{Y}}_{t}\right|}*100\%,$$ (1)

where *k* is the forecasting horizon, *Y*_*t*_ are the actual observations and $\hat{Y_{t}}$ the forecasts produced by the model at point *t*. Since sMAPE penalizes large positive errors more than negative ones [32], the MASE was also introduced to complement the former [31]. This is defined as follows:

$$\begin{array}{c} MASE=\frac{1}{k}\frac{\sum_{t=1}^{k}\left|Y_{t}-{\hat{Y}}_{t}\right|}{\frac{1}{n-m}\sum_{t=m+1}^{n}\left|Y_{t}-Y_{t-m}\right|} \end{array}$$ (2)

where *n* is the number of the available historical observations and *m* is the frequency of the time series. MASE, among its other characteristics, is independent of the scale of the data, its value being less than one if the forecast is more accurate than the average model fitting prediction of the seasonal Naive benchmark, and greater than one if it is less accurate.

We note that, in order to estimate the average forecasting accuracy more indicatively, $\hat{Y_{t}}$ were computed ten times and the average of the errors produced was used to avoid problems caused by the selection of the initial values chosen for the parameterization of the ML methods [33]. In this paper the first *n-18* observations were used for training/validating the models, and the last 18 for testing their forecasting accuracy (following the same procedure as that of the M Competitions).

### 3.2 Computational complexity and model fitting

Computational Complexity (CC) is used to determine the time needed to train a given model and use it for extrapolation. Thus, CC can be simply defined as the mean computational time required by the model to predict a time series, divided by the corresponding time needed by the Naive method to achieve the same task. In this regard, we end up with a relative metric indicating the additional, proportional time required for obtaining the forecasts from the more complex methods. Computational time was estimated using a system with the following characteristics: Intel^®^ Core^™^ i7-4790 CPU @ 3.60GHz, 8.00 GB RAM, x64 based processor.

$$\begin{array}{c} \text{Computational}\;\text{Complexity}(\text{CC})=\frac{\text{Computational}\;\text{Time}\;\text{Model}}{\text{Computational}\;\text{Time}\;\text{Naive}} \end{array}$$ (3)

Finally, the accuracy of how well a model fits (MF) the historical data is defined as

$$\begin{array}{cc} \text{Model}\;\text{Fitting}(\text{MF}) & =\frac{n\sum_{t=1}^{n}{\left(Y_{t}-\hat{Y_{t}}\right)}^{2}}{{\left(\sum_{t=1}^{n}Y_{t}\right)}^{2}}*100\%, \end{array}$$ (4)

Expression (4) is actually the Mean Squared Error (MSE) of the *n-k* model fit forecasts, normalized by the mean value of the time series being examined.

### 3.3 Statistical and ML methods utilized

To compare the performance of ML methods to traditional statistical ones, we included the six most accurate methods of the M3 Competition plus a naive benchmark (the first method), Naive 2, which is actually a random walk model adjusted for seasonality. The second method is Simple Exponential Smoothing (SES) [34], aimed at predicting series without a trend. The third and fourth, Holt and Damped exponential smoothing [22], are most appropriate for time series with trend. The fifth is a combination (average) of the three exponential smoothing methods just described: SES, Holt and Damped (Comb), aimed at achieving the possible benefits of averaging the errors of multiple forecasts [35]. The sixth model is the Theta method [20], which achieved the best overall sMAPE in the original M3 Competition. Finally, the seventh and eighth models are automatic model selection algorithms for ARIMA [36] and exponential smoothing (ETS) [37] models which recent studies have shown to be of considerable accuracy. Moreover, the former serves as a good benchmark to compare ML models, being the linear form of the most popular neural network, the Multi-Layer Perceptron [38]. For an analytical description of the statistical methods used in this study see [39].

A brief description of the same eight ML methods used by Ahmed and co-authors as well as by this study is provided next. Additionally, RNN [40] and LSTM [41] that have recently attracted a lot of interest in the forecasting field, are also included in this study and their accuracy is compared with those of the other methods.

#### 3.3.1 Multi-Layer Perceptron (MLP)

First, a single hidden layer NN is constructed. Then, the best number of input nodes *N* = [1, 2, …, 5] is defined by using a 10-fold validation process, with the inputs being observations *Y*_*t*−5_, *Y*_*t*−4_, *Y*_*t*−3_, *Y*_*t*−2_, and *Y*_*t*−1_ for predicting the time series at point *t*, and doing so for all the *n* − 18 data. Third, the number of the hidden nodes is set to 2*N* + 1 following the practical guidelines suggested by [42] aimed at decreasing the computational time needed for constructing the NN model (the number of the hidden layers used is typically of secondary importance [2]). The Scaled Conjugate Gradient method [43] is then used instead of Standard Backpropagation for estimating the optimal weights. The method, which is an alternative of the famous Levenberg-Marquardt algorithm, has been found to perform better in many applications and is considered more appropriate for weight optimization. The learning rate is selected between 0.1 and 1, using random initial weights for starting the training process with a maximum of 500 iterations. Finally, to maximize the flexibility of the method, although the activation function of the hidden layer is a logistic one, a linear function is used for the output nodes. This is crucial since, if a logistic output activation function is used for optimizing trended time series, it is bounded and “doomed to fail” [28]. In addition, due to the nonlinear activation functions, the data is scaled between 0 to 1 to avoid computational problems, meet algorithm requirement and facilitate faster network learning [2]. The linear transformation is as follows:

$$\begin{array}{c} Y^{\prime }=\frac{Y-Y_{min}}{Y_{max}-Y_{min}} \end{array}$$ (5)

Once all predictions are made, the forecasts are then rescaled back to the original scale.

Having defined the architecture of the optimal neural network, 100 MLP models were additionally trained and used to extrapolate the series. The mean, median and mode of the individual forecasts were then used as the final forecasts. This is done to evaluate the possible benefits of forecast combination, extensively reported in the literature, especially for the case of neural networks which are characterized by great variations when different initial parameters are used [44]. Yet, given that the gains in accuracy for combining multiple forecasts are negligible and the complexity is almost double, we do not present all the results as doing so is unnecessary.

The MLP method is constructed using the *mlp* function of the *RSNNS* R statistical package [45].

#### 3.3.2 Bayesian Neural Network (BNN)

The BNN is similar to the MLP method but optimizes the network parameters according to the Bayesian concept, meaning that the weights are estimated assuming some a priori distributions of errors. The method is constructed according to the suggestions provided by [46] and [47]. The Nguyen and Widrow algorithm [48] is used to assign initial weights and the Gauss-Newton algorithm to perform the optimization. Similar to the MLP method, the best number of input nodes *N* = [1, 2, …, 5] is defined using a 10-fold validation process and the number of the hidden nodes is set to 2*N* + 1. A total number of 500 iterations are considered and the data are linearly scaled.

The BNN method is constructed exploiting the *brnn* function of the *brnn* R statistical package [49].

#### 3.3.3 Radial Basis Functions (RBF)

RBF is a feed-forward network with one hidden layer and is similar to the MLP method. Yet, instead of using a sigmoid activation function, it performs a linear combination of *n* basis functions that are radially symmetric around a center. Thus, information is represented locally in the network, which allows the method to be more interpretable and faster to compute. Like the previous approaches, the best number of input nodes *N* = [1, 2, …, 5] is defined using a 10-fold validation process and the number of the hidden nodes is automatically set to 2*N* + 1. A total number of 500 iterations are considered and the data are linearly scaled. The output activation function is the linear one.

The RBF method is constructed exploiting the *rbf* function of the *RSNNS* R statistical package [45].

#### 3.3.4 Generalized Regression Neural Networks (GRNN)

The GRNN method, also called the Nadaraya-Watson estimator or the kernel regression estimator, is implemented by the algorithm proposed by [50]. In contrast to the previous methods, GRNN is nonparametric and the predictions are found by averaging the target outputs of the training data points according to their distance from the observation provided each time. The sigma parameter, which determines the smoothness of fit, is selected together with the number of the inputs *N* using the 10-fold validation process. The inputs, linearly scaled, vary from 1 to 5 and the sigma from 0.05 to 1, with a step of 0.05.

The GRNN method is constructed exploiting the *guess*, *learn* and *smooth* functions of the *grnn* R statistical package [51].

#### 3.3.5 K-Nearest Neighbor regression (KNN)

KNN is a nonparametric regression method basing its forecasts on a similarity measure, the Euclidean distance between the points used for training and testing the method. Thus, given the *N* inputs, the method picks the closest K training data points and sets the prediction as the average of the target output values for these points. The *K* parameter, which determines the smoothness of fit, is once again optimized together with the number of inputs using the 10-fold validation process. The inputs, which are linearly scaled, may vary from 1 to 5 and the *K* from 2 to 10.

The KNN method is constructed exploiting the *KNN* function of the *class* R statistical package [52].

#### 3.3.6 CART regression trees (CART)

CART is a regression method based on tree-like recursive partitioning of the input space [53]. The space specified by the training sample is divided into regions, called the terminal leaves. Then, a sequence of tests is introduced and applied to decision nodes in order to define in which leave node an object should be classified based on the input provided. The tests are applied serially from the root node to the leaves, until a final decision is made. Like the previous approaches, the total number of input nodes *N* = [1, 2, …, 5] is defined using a 10-fold validation process and are then linearly scaled.

The CART method is constructed exploiting the *rpart* function of the *rpart* R statistical package [54].

#### 3.3.7 Support Vector Regression (SVR)

SVR is the regression process performed by a Support Vector Machine which tries to identify the hyperplane that maximizes the margin between two classes and minimize the total error under tolerance [55]. In order for an efficient SVM to be constructed, a penalty of complexity is also introduced, balancing forecasting accuracy and computational performance. Since in the present study accuracy is far more important than complexity, forecasts were produced using an *ϵ*-regression SVM which maximizes the borders of the margin under suitable conditions to avoid outlier inclusion, allowing the SVM decide the number of the support vectors needed. The kernel used in training and predicting is the radial basic one, mainly due to its good general performance and the few parameters it requires. Following the suggestions of [15], *ϵ* is set equal to the noise level of the training sample, while the cost of constraints violation *C* is fixed to the maximum of the target output values, which is 1. Then, the *γ* parameter is optimized together with the total number of inputs *N* set for the method, using a 10-fold validation process. The inputs are linearly scaled as in the previous methods described.

The SVR method is constructed exploiting the *SVM* function of the *e1071* R statistical package [56].

#### 3.3.8 Gaussian Processes (GP)

According to GP, every target variable can be associated with one or more normally distributed random variables which form a multivariate normal distribution, emerging by combining the individual distributions of the independent ones [57]. In this respect, Gaussian processes can serve as a nonparametric regression method which assumes an a priori distribution for the input variables provided during training, and then combines them appropriately using a measure of similarity between points (the kernel function) to predict the future value of the variable of interest. In our case, the input variables are the past observations of the time series, linearly scaled, while their total number *N* = [1, 2, …, 5] is defined using a 10-fold validation process. The kernel function used is the radial basis one, while the initial noise variance and the tolerance of termination was set to 0.001 given that, as suggested by [15], it would be computationally prohibitive to use a three-dimensional 10-fold validation approach to define them.

The GP method is constructed exploiting the *gausspr* function of the *kernlab* R statistical package [58].

#### 3.3.9 Recurrent Neural Network (RNN)

Simple RNN, also known as Elman network [59], has a similar structure to the MLP, but contains feedback connections in order to take into account previous states along with the current input before producing the final output(s). This is done by saving a copy of the previous values of the layer containing the recurrent nodes and using them as an additional input for the next step. In this respect, the network is allowed to exhibit dynamic temporal behavior for a time sequence.

In this study, the model used to implement the RNN is the sequential one. It is comprised of two layers, a hidden one containing recurrent nodes and an output one containing one or more linear nodes. Due to high computational requirements, we did not use k-fold validation for choosing the optimal network architecture per series but rather three input nodes and six recurrent units, forming the hidden layer, for all the time series of the dataset. The selection was made based on the results of a random sample of series for which this parameterization displayed the best performance. Regarding the rest of the hyper-parameters, a number of 500 epochs was chosen and the learning ratio was set to 0.001, with the linear activation function being used in all nodes.

The RNN method was constructed exploiting the SimpleRNN and Dense class of the Keras API v2.0.9 [60] for Python 2.7 among with TensorFlow framework v1.4.0 [61].

#### 3.3.10 Long Short Term Memory neural network (LSTM)

The LSTM network is similar to the RNN discussed above and was proposed by [41] to avoid the long-term dependency problem present for the case of the latter. The advantage of LSTM units have over regular RNN units is their ability to keep information over longer periods of time due to their complex architecture which consists of several gates with the power to remove or add information to the unit’s state.

Similar to RNN, the model used to implement the LSTM network is the sequential one comprised of a hidden and an output layer. Similarly, due to high computational time, the architecture of the model consists of three input nodes, six LSTM units forming the hidden layer and a single linear node in the output layer. The linear activation function is used before the output of all units and the hard sigmoid one for the recurrent step. Regarding the rest of the hyper-parameters, the rmsprop optimizer was used, a number of 500 epochs was chosen and the learning ratio was set to 0.001.

The RNN method was constructed exploiting the LSTM and Dense class of the Keras API v2.0.9 [60] for Python 2.7 among with TensorFlow framework v1.4.0 [61].

### 3.4 Data preprocessing

In contrast to sophisticated time series forecasting methods, where achieving stationarity in both the mean and variance is considered essential, the literature of ML is divided with some studies claiming that ML methods are capable of effectively modelling any type of data pattern and can therefore be applied to the original data [62]. Other studies however, have concluded the opposite, claiming that without appropriate preprocessing, ML methods may become unstable and yield suboptimal results [28].

Preprocessing can be applied in three forms: Seasonal adjustments, log or power transformations, and removing the trend. For instance, [11] found that MLP cannot capture seasonality adequately, while [63] claim exactly the opposite. There is greater agreement concerning the trend, given the bouncing of MLP’s activation function that become more stable once the series has been detrended [64]. Yet, more empirical results are needed to support the conclusions related to preprocessing, including the most appropriate way to eliminate the trend in the data [65]. In this study, the following indicative alternatives are used:

- **Original data**: No pre-processing is applied.
- **Transforming the data**: The log or the Box-Cox [66] power transformation is applied to the original data in order to achieve stationarity in the variance.
- **Deseasonalizing the data**: The data is considered seasonal if a significant autocorrelation coefficient at lag 12 exists. In such case the data is deseasonalized using the classical, multiplicative decomposition approach [39]. The training of the ML weights, or the optimization of statistical methods, is subsequently done on the seasonally adjusted data. The forecasts obtained are then reseasonalized to determine the final predictions. This is not done in the case of ETS and ARIMA methods since they include seasonal models, selected using relative tests and information criteria that take care of seasonality and model complexity directly.
- **Detrending the data**: A Cox-Stuart test [67] is performed to establish whether a deterministic linear trend should be used, or alternatively first differencing, to eliminate the trend from the data and achieve stationarity in the mean.
- **Combination of the above three**: The benefits of individual preprocessing techniques are applied simultaneously to adjust the original data.

In order to speed up computations, we first determine the best preprocessing alternative for improving the post-sample one-step-ahead forecasting performance of the MLP method (the most popular among ML ones) and then apply that one to the remaining ML models. Table 4 summarizes the results of the various preprocessing possibilities as well as their combinations when predicting the 18 unknown observations of the 1045 time series, as applied in the study of [15]. The best combination according to sMAPE is number 7 (Box-Cox transformation, deseasonalization) while the best one according to MASE is number 10 (Box-Cox transformation, deseasonalization and detrending). Some interesting observations from studying the results of Table 4 are the following:

- Transforming the data with the Box-Cox method is a slightly better option than the alternative of using logs in terms of the sMAPE. But both alternatives do not improve its accuracy much and do not provide any improvement in MASE.
- Seasonal adjustments provide significantly better results in both sMAPE and MASE.
- Removing the trend from the data using a linear function provides more accurate results (in both the sMAPE and MASE) than the alternative of using the first difference.
- There are important differences in sMAPE and MASE with each type of preprocessing.

**Table 4:** Forecasting performance of MLP for one-step-ahead forecasts having applied various preprocessing alternatives.

Approach	sMAPE(%)	MASE	CC	MF
**Original data**	9.15	0.67	90.24	2.80
**Transformation: Log**	8.99	0.67	88.04	3.06
**Transformation: Box-Cox**	8.97	0.67	88.07	2.99
**Detrending: Linear deterministic function**	10.43	0.65	87.05	2.67
**Detrending: First differencing**	11.87	0.86	85.02	2.95
**Deseasonalisation**	8.16	0.57	93.31	2.10
**Combination 3&6**	**7.96**	0.56	88.54	2.16
**Combination 6&4**	9.56	0.56	84.44	**2.01**
**Combination 3&4**	9.07	0.64	88.78	2.82
**Combination 3&6&4**	8.39	**0.55**	**83.49**	2.11

The bold numbers highlight the best performing approach per metric.

More work on the most appropriate preprocessing approach is definitely required, first to confirm the results found using a subset of the series of the M3 Competition and second to determine if the conclusions made using the MLP method will be similar to those of other ML ones.

Transforming the data also seems beneficial when dealing with traditional forecasting methods. This can be seen by examining Tables 5 and 6, that display the forecasting performance for the eight statistical methods included in this work according to sMAPE and MASE. The sMAPE accuracies show a consistent improvement, while those of MASE are about the same. Moreover, after transformations, the differences between the various methods become smaller, meaning that simpler methods, such as Damped, can now be used instead of ETS, which may be more accurate but also the most time intensive.

**Table 5:** Forecasting performance of the eight statistical methods included in the study for one-step-ahead forecasts using the original data.

Method	sMAPE(%)	MASE	CC	MF
**Naive 2**	8.59	0.56	1.00	3.63
**SES**	7.36	0.49	1.53	2.37
**Holt**	7.41	0.48	2.31	2.35
**Damped**	7.30	0.48	3.96	2.34
**Comb**	7.27	0.48	6.88	2.32
**Theta**	7.31	0.48	5.84	2.34
**ARIMA**	7.34	0.47	43.96	2.53
**ETS**	7.19	0.47	34.07	2.28

**Table 6:** Forecasting performance of the eight statistical methods included in the study for one-step-ahead forecasts having applied the Box-Cox transformation.

Method	sMAPE(%)	MASE	CC	MF
**Naive 2**	8.58	0.56	1.28	3.66
**SES**	7.25	0.49	1.69	2.38
**Holt**	7.32	0.48	2.45	2.35
**Damped**	7.19	0.48	4.54	2.33
**Comb**	7.20	0.48	7.23	2.32
**Theta**	7.23	0.48	5.75	2.36
**ARIMA**	7.19	0.47	46.56	2.59
**ETS**	7.12	0.47	35.55	2.30

Table 7 compares the one-step-ahead forecasts of the ML methods used by Ahmed and colleagues and by our own study once the most appropriate preprocessing has been applied. This includes the Box-Cox transformation, deseasonalization and detrending since evaluating forecasting performance through MASE instead of sMAPE is considered to be, as mentioned in section 3.1, a more reliable choice. There are some important similarities in the overall sMAPE (see column 2) between the results of [15] and our own indicating that the forecasts of ML methods are consistent over the period of seven years. There are also some important differences that can be justified given the length of time that has passed between the two studies and the computational advancements in utilizing the ML algorithms. At the same time, the reasons for the huge difference in RBF (9.57% vs 15.79%) as well as the smaller ones in CART and SVR need to be investigated. Undoubtedly, the use of slightly different parameterisation for applying the individual methods, as well as the exploitation of different functions for their implementation (R instead of Matlab) might explain part of the variations. It should be noted that RNN is among the less accurate ML methods, demonstrating that research progress does not necessarily guarantee improvements in forecasting performance. This conclusion also applies in the performance of LSTM, another popular and more advanced ML method, which does not enhance forecasting accuracy too.

**Table 7:** Forecasting performance of the ten ML methods included in the study for one-step-ahead forecasts having applied the most appropriate preprocessing alternative.

Method	sMAPE(%)	MASE	CC	MF
**MLP**	8.39	0.55	83.49	2.11
**MLP (Ahmed et al.)**	8.34			
**BNN**	8.17	0.53	47.44	2.11
**BNN (Ahmed et al.)**	8.56			
**RBF**	9.57	0.71	146.11	1.66
**RBF (Ahmed et al.)**	15.79			
**GRNN**	9.49	0.67	388.73	1.80
**GRNN (Ahmed et al.)**	10.33			
**KNN**	11.49	0.80	12.01	3.30
**KNN (Ahmed et al.)**	10.34			
**CART**	10.28	0.74	8.89	1.74
**CART (Ahmed et al.)**	11.72			
**SVR**	8.88	0.61	9.79	2.11
**SVR (Ahmed et al.)**	10.40			
**GP**	9.14	0.62	29.39	2.09
**GP (Ahmed et al.)**	9.62			
**RNN**	9.48	0.54	23.18	1.98
**LSTM**	11.67	0.72	48.66	1.84

The corresponding accuracies of Ahmed and coauthors, from their Table 3 p. 611 are also shown for reasons of comparison.

The results in Table 7 show that MLP and BNN outperform the remaining ML methods. Thus, these two are the only ones to be further investigated by comparing their forecasting accuracy beyond one-step-ahead predictions to multiple horizons, useful for those interested in predicting beyond one horizon.

### 3.5 Multiple-horizon forecasts

There are three alternative ways for producing multiple period forecasts with ML models. Given that it is not possible to claim that one is superior for all forecasting situations [3], all of them are considered.

#### 3.5.1 Iterative forecasting

The first alternative is found in the exact same way as the one-step-ahead approach used by Ahmed et al. and by our own study. It is possible, however, to obtain forecasts additionally to one-step-ahead using the first forecast produced by the model, instead of the actual value, to get a forecast for horizon two and then use the first two forecasts to estimate the one for horizon 3 and so on, until predictions for all 18 horizons have been found (this is also the approach used by the ARIMA models in the M Competitions). This means that we obtain 18 forecasts using the first n-18 data points only. As the forecasting horizon increases, the new forecasts depend on the accuracy of the previous ones, meaning that longer term ones may deteriorate. The advantage of this approach is its simplicity and computational ease.

#### 3.5.2 Direct forecasting

The direct approach produces multiple-step-ahead forecasts, instead of one-step-ahead ones, by training and exploiting an 18-output-node neural network able of producing 18 forecasts simultaneously, one for each forecasting horizon. The first output node is responsible for forecasting horizon h = 1, output node 2 for forecasting horizon h = 2, ending with output mode 18 for forecasting horizon 18. It will be interesting to determine if this multi-forecast approach will be more accurate than the iterative one, knowing well that the Direct approach is more complex and computationally more demanding, while fewer observations are available for training.

#### 3.5.3 Multi-neural network forecasting

**Fig 1:**
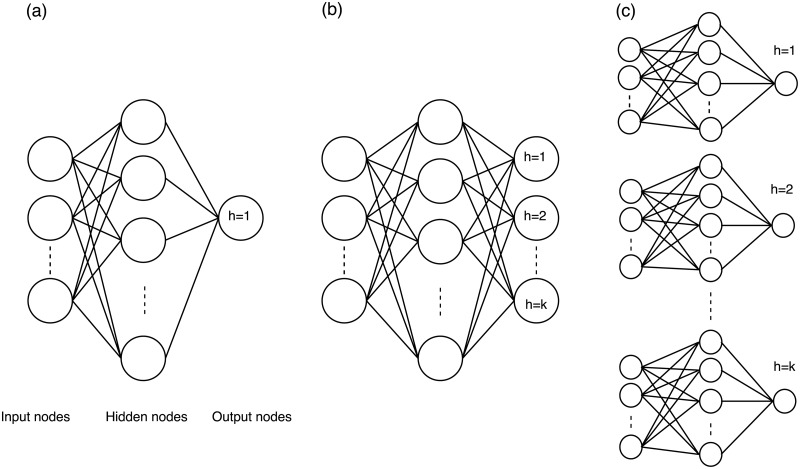
The three possible multi-step-ahead forecasting approaches used by NNs. (a) The iterative, (b) the direct and (c) the multi-neural network forecasting approach.

In this approach, single output node NNs are trained for producing forecasts. Yet, instead of training one NN for each horizon simultaneously, 18 separate NNs are trained, each one for predicting a single h-step-ahead forecast. In this respect, if we wish to forecast the value of the time series for one horizon-ahead we use the first NN trained using the n-18 data, for two the second NN again trained in the n-18 data, and so on for eighteen times in total. The three options for multi-horizon forecasts can be visualized in Fig 1.

The results of each of the three approaches for predicting 18 months ahead are displayed in Tables 8 and 9 for both the ML methods as well as the eight statistical ones using sMAPE and MASE. To simplify the presentation, the results are grouped into three forecasting horizons: Short-term (1 to 6 months ahead), Medium-term (7 to 12 months ahead) and, finally, Long-term (13 to 18 months ahead), while the accuracies for all horizons can be found in Tables A1 and A2 in the Appendix at the end of this paper. Tables 8 and 9 as well as A1 and A2 allow us to evaluate the accuracy achieved by each method across multiple horizons and decide on their appropriateness for various applications. We also note that for the case of the BNN, the direct approach was excluded since it is not supported by the *brrn* function exploited for obtaining the forecasts in our study.

**Table 8:** Forecasting performance (sMAPE) of ML and Statistical methods across various horizons having applied the most appropriate preprocessing alternative.

Method	Short	Medium	Long	Average	CC
**MLP Iterative**	9.53	12.34	15.00	12.29	245.58
**MLP Direct**	10.72	13.55	16.20	13.49	438.53
**MLP Multi**	9.53	12.69	16.08	12.77	4006.82
**BNN Iterative**	9.39	12.08	14.80	12.09	141.91
**BNN Multi**	9.48	12.70	15.96	12.71	2046.49
**Naive 2**	10.78	12.46	15.08	12.77	**1.48**
**SES**	9.17	10.85	13.77	11.26	1.60
**Holt**	9.07	11.18	14.29	11.51	1.75
**Damped**	8.96	10.63	13.46	11.02	2.07
**Comb**	8.95	10.57	13.38	10.97	2.65
**Theta**	8.96	**10.53**	**13.19**	**10.89**	1.70
**ARIMA**	**8.93**	11.08	13.84	11.28	73.50
**ETS**	9.07	10.98	13.74	11.26	56.66

The bold numbers highlight the best performing method per forecasting horizon and computational complexity.

**Table 9:** Forecasting performance (MASE) of ML and statistical methods across various horizons having applied the most appropriate preprocessing alternative.

Method	Short	Medium	Long	Average	CC
**MLP Iterative**	0.66	0.98	1.24	0.96	245.58
**MLP Direct**	0.76	1.10	1.38	1.08	438.53
**MLP Multi**	0.65	1.02	1.37	1.01	4006.82
**BNN Iterative**	0.64	0.94	1.20	0.93	141.91
**BNN Multi**	0.65	1.02	1.35	1.01	2046.49
**Naive 2**	0.76	1.05	1.35	1.05	**1.48**
**SES**	0.67	0.96	1.29	0.97	1.60
**Holt**	0.64	0.92	1.25	0.94	1.75
**Damped**	0.64	0.91	1.21	0.92	2.07
**Comb**	0.64	0.902	1.20	0.91	2.65
**Theta**	0.64	**0.89**	**1.17**	0.90	1.70
**ARIMA**	**0.61**	0.89	1.17	**0.89**	73.50
**ETS**	0.64	0.92	1.21	0.92	56.66

The bold numbers highlight the best performing method per forecasting horizon and computational complexity.

In brief, statistical models seem to generally outperform ML methods across all forecasting horizons, with Theta, Comb and ARIMA being the dominant ones among the competitors according to both error metrics examined. It is also notable that good forecasting accuracy comes with great efficiency, meaning that CC is not significantly increased for the best performing methods. It is also worth mentioning that more complex approaches of extrapolation through ML methods, such as the direct and Multi one, display less accurate results indicating that complex is not always better and that ML methods fail to learn how to best predict for each individual forecasting horizon.

## 4 The accuracy, the goodness of fit and the computational complexity of the ML methods

**Fig 2:**
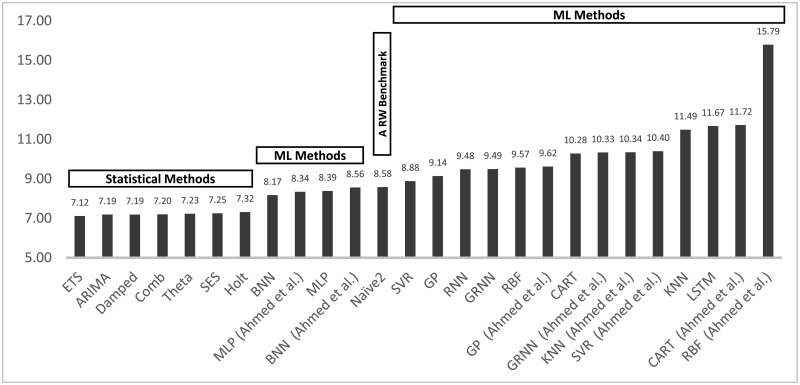
Forecasting performance (sMAPE) of the ML and statistical methods included in the study. The results are reported for one-step-ahead forecasts having applied the most appropriate preprocessing alternative.

Fig 2 shows the overall sMAPE for all the statistical and ML methods included in this paper as well as the ML accuracies reported by Ahmed and colleagues for performing one-step-ahead forecasts. As seen, the six most accurate methods are statistical, confirming their dominance over the ML ones. Even Naive 2 (a seasonal Random Walk (RW) benchmark) is more accurate than half of the ML methods. The most interesting question and greatest challenge is to find the reasons for their poor performance with the objective of improving their accuracy and exploiting their huge potential. AI learning algorithms have revolutionized a wide range of applications in diverse fields and there is no reason that the same cannot be achieved with the ML methods in forecasting. Thus, we must find how to be applied to improve their ability to forecast more accurately.

ML models are nonlinear functions connecting the inputs and outputs of neurons. The goal of the network is to “learn” by solving an optimization problem in order to choose a set of parameters, or weights, that minimize an error function, typically the sum of square errors. However, the same type of optimization is done in ARIMA (or regression) models. There is no obvious reason, therefore, to justify the more than 1.24% higher sMAPE of MLP, one of the best ML methods, in comparison to that of ARIMA, or that the sMAPE of this MLP is only 0.19% more accurate than Naive 2, the seasonally adjusted random walk model. Respectively, one would expect RNN and LSTM, which are more advanced types of NNs, to be far more accurate than the ARIMA and the rest of the statistical methods utilized. Clearly, if there was any form of learning, the accuracy of ML methods should have exceeded that of ARIMA and greatly outperform the Naive 2. Thus, it is imperative to investigate the reasons that this is not happening, e.g. by comparing the accuracy of ML and statistical methods series by series, explaining the differences observed and identifying the reasons involved.

The more serious issue, simply put, is how ML methods can be made to learn about the unknown future rather how well a model fits past data. For this to be done, the ML methods must have access to information about the future and their objective must be to minimize future errors rather than those of fitting a model to available data. Until a later time when more advanced ML methods become available and in order to simplify things, we suggest that the data is deseasonalized before some ML model is utilized, as research [11] has shown little to no differences between the post-sample accuracy of models applied to original and seasonally adjusted data.

A practical way to allow learning about the unknown future errors is by dividing the *n* − 18 data into two parts, with the first one containing the 1/3 of the *n* − 18 data and the second the remaining 2/3. If the data is first deseasonalized, a much simpler model can be developed using the first (*n* − 18)/3 data and then trained to learn how to best predict the next 18 observations. Then, the first (*n* − 18)/3 + 1 data can be used to let the method learn how to best predict the next 18 observations and continue using the first (*n* − 18)/3 + 2, the first (*n* − 18)/3 + 3 and so on, until having used all the observations available. Clearly, such a sliding simulation, attempting to predict future values based on post-sample accuracy optimization, will probably be a step in the right direction even though its performance needs to be empirically tested.

Another possibility is to provide ML methods with alternative forecasts (e.g. the ones produced by the best statistical methods) and ask them to learn to select the most accurate one (or their combination) for each forecasting horizon and series in such a way as to minimize post-sample errors. This may require clustering the data into various categories (micro, macro, demographic etc.) or types of series (seasonal/non-seasonal, trended/non-trended, of high, medium or low randomness etc.) and develop different models for each category/type. In Table 6 of [15], for instance, accuracy varies significantly depending on the category of the series with the best one being in demographic and macro data, the worst in micro and industry time series, and finance in between. This may indicate that ML methods could under-perform among others, due to the fact that they are confused when attempting to optimize specific or heterogeneous data patterns.

An additional concern could be the extent of randomness in the series and the ability of ML models to distinguish the patterns from the noise of the data, avoiding over-fitting. This can be a challenging problem since, in contrast to linear statistical methods, where over-fitting can be directly controlled by some information criteria (e.g., the AIC [68]) taking into account the number of parameters utilized, ML methods are nonlinear and training is performed dynamically, meaning that different forecasts may arise according e.g. to the maximum iterations considered, even if the complexity of the network’s architecture is identical. Since the importance of possible over-fitting by ML methods is critical, the topic will be covered in detail on its own in section 4.1 below.

A final concern with ML methods could be the need for preprocessing that requires individual attention to select the most appropriate transformation, possible deseasonalization, as well as trend removal. Effective ML methods must, however, be able to learn and decide on their own the most appropriate preprocessing as there are few possibilities available. If, for example, the Box-Cox criterion can be used to determine the most appropriate transformation for statistical methods, it makes no sense that something similar cannot be applied by ML methods to automate preprocessing, simplify the modeling process and probably improve accuracy by doing so.

### 4.1 Over-fitting

Tables 4, 5 and 6 report, among others, the goodness of fit, indicating how well the trained model fitted the n-18 observations available for each series. Yet, model fit is not a good predictor of post-sample forecasting accuracy, meaning that methods with low fitting errors might result in higher post-sample ones and vice versa. One would expect for instance that the MLP method, displaying a model fitting error of 2.11%, would forecast more accurately than the ARIMA whose corresponding error is higher (2.59%). However, this is not the case as the post-sample sMAPE of the two methods are 8.39% and 7.19%, respectively. Moreover, RBF, GRNN and CART, which have the best model fitting, are some of the worst performing methods. A possible reason for the improved accuracy of the ARIMA models is that their parameterization is done through the minimization of the AIC criterion, which avoids over-fitting by considering both goodness of fit and model complexity. In contrast, the MLP method specifies its complexity (input nodes) through cross-validation, but no additional criteria are applied for mitigating over-fitting e.g. by specifying when training should stop. The maximum number of iterations defined serves that purpose, yet there is no global optima: in some time series, over-fit might occur after a few iterations, while in others after many hundreds.

**Fig 3:**
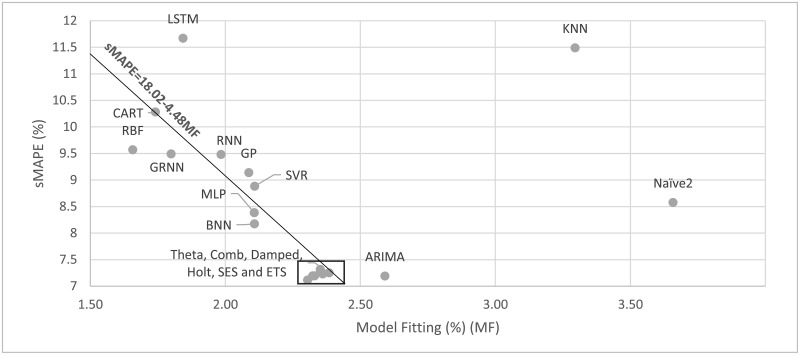
Forecasting performance (sMAPE) versus model fitting. The results are reported for one-step-ahead forecasts having applied the most appropriate preprocessing alternative.

Fig 3 shows the sMAPE (vertical axis) and the accuracy of model fit (horizontal axis). It is clear from this Fig that the old belief that minimizing the model fit errors would guarantee more accurate post-sample predictions does not hold, and that some criteria similar to the AIC or other successful techniques [69] would be required to indicate to ML methods when to stop the optimization process and avoid considering as pattern a part of the noise of the data. In our view, considerable improvements can result by such an action.

### 4.2 Computational complexity

**Fig 4:**
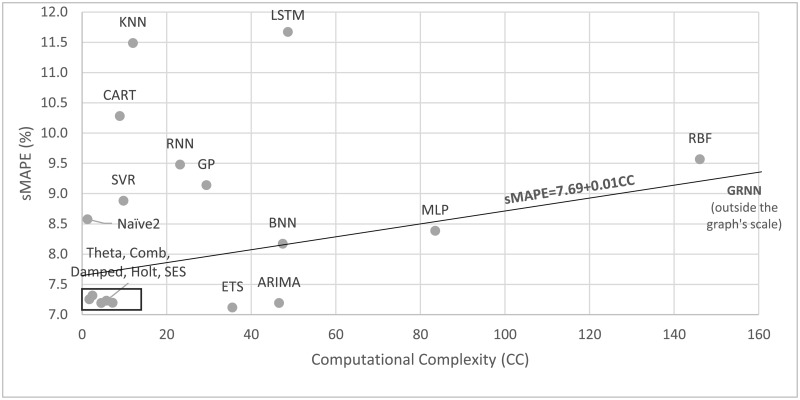
Forecasting performance (sMAPE) versus computational complexity. The results are reported for one-step-ahead forecasts having applied the most appropriate preprocessing alternative.

As forecasting methods are used in various applications, the computational time required to forecast becomes critical. It would be impractical for example to utilize the ML GRNN method (the most computationally demanding) to predict the demand for hundreds of thousands of inventory items, even though computers are becoming faster and cheaper. Memory and CPU usage optimization might serve in that direction but again, computational intensity remains an important issue. For instance, despite exploiting such optimization processes in our study, reducing the computational time of the ML methods by more than 30%, the complexity reported is still much greater compared to the statistical ones. For this reason, the information provided in Fig 4 is of value, as it confirms the low computational requirements of statistical methods, lying in the lower left part of the Fig, and additionally shows that superior accuracy can be achieved with less computational effort. In particular, the five inside the square box (Damped, Comb, Theta, SES and Holt) are not only some of the most accurate but also—apart from ETS—the least computationally demanding.

For practical reasons, if ML methods are to be applied by business and non-profit organizations, their computational requirements must be reduced considerably. This can be done by deseasonalizing the data first, utilizing simpler models, limiting the number of training iterations or choosing the initial values of the weights, not in a completely arbitrary manner, but through a guided search that would provide values not too far from the optimal ones. Alternatively, the speed of moving towards the optimal can increase in order to reduce the computational time to reach such an optimal. These improvements would require testing to determine the trade-offs between lesser accuracy, resulting from the reduction in computational time, versus the savings from such a reduction.

## 5 Conclusions: The state of the art and ways forward

The field of statistical forecasting has progressed a great deal since the early dates when [70] used exponential smoothing, in the late 1940s, for predicting the inventory demand for many thousands of items in navy shipyards. The introduction of the Box-Jenkins methodology to ARIMA models [71] brought academic respectability to a field dominated until then by practitioners, while the extensive use of regression and econometric models [72] further enlarged the field. Finally, multivariate GARCH models were also made available [73, 74] broadening the coverage of the field (for an excellent survey of the latest developments see Special Issue on “Simple Versus Complex Forecasting” [75]).

A major innovation that has distinguished forecasting from other fields has been the good number of empirical studies aimed at both the academic community as well as the practitioners interested in utilizing the most accurate methods for their various applications and reducing cost or maximizing benefits by doing so. These studies contributed to establishing two major changes in the attitudes towards forecasting: First, it was established that methods or models, that best fitted available data, did not necessarily result in more accurate post sample predictions (a common belief until then). Second, the post-sample predictions of simple statistical methods were found to be at least as accurate as the sophisticated ones. This finding was furiously objected to by theoretical statisticians [76], who claimed that a simple method being a special case of e.g. ARIMA models, could not be more accurate than the ARIMA one, refusing to accept the empirical evidence proving the opposite. These two findings have fundamentally changed the field of forecasting and are also evident in this paper both in Fig 3, showing post-sample versus in-sample accuracy, as well as in Fig 2, displaying the accuracy level of various statistical and ML methods, with the latter being much more sophisticated and computationally demanding than the former.

Knowing that a certain sophisticated method is not as accurate as a much simpler one is upsetting from a scientific point of view as the former requires a great deal of academic expertise and ample computer time to be applied. At the same time, understanding the reasons of their underperformance is the only way to improve them. This has certainly been the case with ARIMA models whose accuracy with monthly data (not the same as those used in this study) in the 1982 M Competition was 17.9% and has decreased to 11.28% in the present study, tying with the accuracy of the damped exponential smoothing, one of the most accurate methods of the M Competitions. ARIMA’s improved performance is mainly due to the utilization of the AIC criterion and other optimization processes, enabling effective automatic model selection and parameterization, while avoiding or minimizing over-fitting. Another interesting example could be the case of LSTM that compared to simpler NNs like RNN and MLP, report better model fitting but worse forecasting accuracy.

ML theorists working on forecasting applications need to do something to improve the accuracy of their methods. For instance, the only thing exponential smoothing methods do is smoothen the most recent errors exponentially and then extrapolate the latest pattern in order to forecast. Given their ability to learn, ML methods should do better than simple benchmarks, like exponential smoothing. Accepting the problem is the first step in devising workable solutions and we hope that those in the field of AI and ML will accept the empirical findings and work to improve the forecasting accuracy of their methods.

A problem with the academic ML forecasting literature is that the majority of published studies provide forecasts and claim satisfactory accuracies without comparing them with simple statistical methods or even naive benchmarks. Doing so raises expectations that ML methods provide accurate predictions, but without any empirical proof that this is the case. In our view, this situation is the same with what was happening in statistical literature in the late 1970s and 1980s. At that time, it was thought that forecasting methods were of superior accuracy simply because of their sophistication and their mathematical elegance. Now it is obvious that their value must be empirically proven in an objective, indisputable manner through large scale competitions. Thus, when it comes to papers proposing new ML methods, or effective ways to use them, academic journals must demand comparisons with alternative methods or at least benchmarks and require that the data of the articles being published be made available for those who want to replicate the results. In our experience, this has not been the case at present, making replications practically impossible and allowing conclusions that may not hold.

In addition to empirical testing, research work is needed to help users understand how the forecasts of ML methods are generated (this is the same problem with all AI models whose output cannot be explained). Obtaining numbers from a black box is not acceptable to practitioners who need to know how forecasts arise and how they can be influenced or adjusted to arrive at workable predictions.

A final, equally important concern is that in addition to point forecasts, ML methods must also be capable of specifying the uncertainty around them, or alternatively providing confidence intervals. At present, the issue of uncertainty has not been included in the research agenda of the ML field, leaving a huge vacuum that must be filled as estimating the uncertainty in future predictions is as important as the forecasts themselves. To overcome this issue, many researchers propose simulating the intervals by iteratively generating multiple future sample paths. Yet, even in that case, the forecast distribution of the methods is empirically and not analytically derived, raising many doubts about its quality.

To summarize, according the results of this study, ML methods need to become more accurate, requiring less computer time, and be less of a black box. A major contribution of this paper is in showing that traditional statistical methods are more accurate than ML ones and pointing out the need to discover the reasons involved, as well as devising ways to reverse the situation. However, in the comparisons of the statistical and ML methods reported in this paper, it must be made clear that the results may be related to the specific data set being used. The 3003 time series of M3 come mainly from the business and economic world that seem to be represented truthfully by this data [77], characterized by considerable seasonality, some trend and a fair amount of randomness [78]. The frequency of close to half of the series is monthly, followed by quarterly and yearly ones of about the same percentage. The length of the series varies from 14 for yearly data to 126 for monthly ones, with the majority being in the Micro and Macro domain. The characteristics of the series as well as their length may be a critical factor determining the accuracy of the various methods reported in this paper, requiring additional research, using other data sets, to verify that similar results will hold true if different sets of data are used and, most importantly, the series are of much longer length so the ML methods can train their weights more optimally.

For instance, in [78], the authors use a set of six features to analyze the M3 data, visualizing them in a 2-dimensional space and examining the strengths and weaknesses of different forecasting methods using the new classification. Their results show that the particularities of the dataset may affect the conclusions drawn, indicating that different ones could have emerged if another sample of time series had been selected instead. The relation between forecasting accuracy and time series characteristics is also reported by [79] who claim that there are indeed “Horses for Courses” in demand forecasting. In this regard, even though M3 might be representative of the reality when it comes to business applications, the findings may be different if nonlinear components are present, or if the data is being dominated by other factors. In such cases, the highly flexible ML methods could offer significant advantage over statistical ones. Furthermore, the length of business series, which is relatively limited compared to those of other applications that ML methods are typically utilized (e.g., energy forecasting), may also affect their performance as proper training may be difficult or even impossible when short series are involved. Thus, the conclusions of future studies would be necessary to come up with definite conclusions.

At this point, the following suggestions/speculations, that must be empirically verified, can be made about the way forward regarding the ML methods, while these can be enriched by future research topics proposed in relative surveys [80]:

- Obtain more information about the unknown future values of the data rather than their past ones and base the optimization/learning on such future values as much as possible.
- Deseasonalize the data before using ML methods. This will result to a simpler one, reducing the computational time required to arrive at optimal weights and, therefore, learn faster.
- Use a sliding simulation approach to gain as much information as possible about future values and the resulting uncertainty and learn more effectively how to minimize them.
- Cluster the series into various homogeneous categories and/or types of data and developing ML methods that optimally extrapolate them.
- Avoid over-fitting as it is not clear if ML models can correctly distinguish the noise from the pattern of the data.
- Automate preprocessing and avoid the extra decisions required from the part of the user.
- Allow the estimation of uncertainty for the point forecasts and provide information for the construction of confidence intervals around such forecasts.

Although the conclusion of our paper that the forecasting accuracy of ML models is lower to that of statistical methods may seem disappointing, we are extremely positive about the great potential of ML ones for forecasting applications. Clearly, more work is needed to improve such methods but the same has been the case with all new techniques, including the complex forecasting methods that have improved their accuracy considerably over time. Who could have believed even ten years ago that we will have AVs, personal assistance on our mobile phones understanding and speaking in natural languages, automatic translations in Skype, AlphaGo beating the world GO champion or facial expression recognition algorithms [81]. There is no reason that the same type of breakthroughs cannot be achieved with ML methods applied to forecasting. Even though, we must realize that applying AI to forecasting is quite different than doing so in games or in image and speech recognition and may require different, specialized algorithms to be successful. In contrast to other applications, the future is never identical to the past and training of AI methods cannot exclusively depend on it.

Table 10 is our attempt to show that not all applications can be modeled equally well using AI algorithms. Games are the easiest as the rules are known and do not change, the environment is also known and stable, the predictions cannot influence the future and there is no uncertainty. The exact opposite is true for forecasting applications where not only the rules are not known but can also change, there are structural instabilities in the data, while there is plenty of uncertainty and noise, that can confuse the search for the optimal weights. Moreover, in certain applications, the forecasts themselves can influence, or even change the future creating self-fulfilling or self-defeating prophesies, expanding the level of noise and increasing the level of uncertainty. It may be necessary, therefore, to adapt the algorithms to these conditions and make sure that there is no over-fitting. Judging from the results of this paper, it may be necessary that ML algorithms applied to forecasting may require additional research to experiment with innovative ideas and come up with adjustments in order to achieve more accurate predictions.

**Table 10:** Features of various Artificial Intelligence (AI) applications.

Type of Application	Rules are known and do not change	The environment is known and stable	Predictions can influence the future	Extent of Uncertainty (or amount of noise)	Examples
Games	Yes	Yes	No	None	Chess, GO
Image and speech recognition	Yes	Yes	No	Minimal (can be minimized by big data)	Face Recognition, Siri, Cortana, Google AI
Predictions based on the Law of large numbers	Yes	Yes	Minimally	Measurable (Normally distributed)	Forecasting the sales of beer, coffee, soft drinks, weather etc.
Autonomous Functions	Yes	Yes	No	Can be assessed and minimized	Self-Driving Vehicles
Strategy, Competition, Investments	No	No	Yes, often to a great extent	Cannot be measured (fat tails)	Decisions, Anticipations, Forecasts
Combinations of the above	It may be the ultimate challenge moving towards GAI (General AI) but also increasing the level of complexity and sophistication of algorithms				Eventually it can cover everything

As the first step towards providing a more diverse, large and representative dataset for evaluating the performance of forecasting methods, establishing reliable benchmarks and promoting future research, we have started the M4-Competition (see https://www.m4.unic.ac.cy/) that seeks to identify the most accurate forecasting method(s) for different types of predictions. It aims to compare **all** major time series methods and identify the most appropriate methods for each case. M4 utilizes 100,000 real-life series and has attracted great interest by both academic researchers and practitioners, providing objective evidence of the most appropriate way of forecasting various variables of interest. The new M4 Competition will extend and replicate the results of the previous three ones, while also avoiding the possible problems of M3. Furthermore, it will increase the number of series to 100,000, include additional frequencies, while also augmenting their length considerably. Given the great number of series, it will be possible to utilize advanced data analytics and related technologies to determine the influence of the various factors on forecasting accuracy, as well as to determine the most appropriate methods for different forecasting applications.

## Supporting information

S1 Appendix Containing tables A1 and A2, presenting the analytical results of the forecasting models used in the present study. The accuracy is evaluated per forecasting horizon first according to sMAPE, and then to MASE.
(PDF)

## References

[1] Makridakis S. The forthcoming Artificial Intelligence (AI) revolution: Its impact on society and firms. Futures. 2017;90:46–60. 10.1016/j.futures.2017.03.006

[2] Zhang G, Eddy Patuwo B, Hu Y M. Forecasting with artificial neural networks:: The state of the art. International Journal of Forecasting. 1998;14(1):35–62. 10.1016/S0169-2070(97)00044-7

[3] Hamzaçebi C, Akay D, Kutay F. Comparison of direct and iterative artificial neural network forecast approaches in multi-periodic time series forecasting. Expert Systems with Applications. 2009;36(2, Part 2):3839–3844. 10.1016/j.eswa.2008.02.042.

[4] Deng L. A tutorial survey of architectures, algorithms, and applications for deep learning—ERRATUM. APSIPA Transactions on Signal and Information Processing. 2014;3 10.1017/atsip.2013.9

[5] Zhang L, Suganthan PN. A survey of randomized algorithms for training neural networks. Information Sciences. 2016;364–365(Supplement C):146–155. 10.1016/j.ins.2016.01.039.

[6] Salaken SM, Khosravi A, Nguyen T, Nahavandi S. Extreme learning machine based transfer learning algorithms: A survey. Neurocomputing. 2017;267:516–524. 10.1016/j.neucom.2017.06.037.

[7] Robinson C, Dilkina B, Hubbs J, Zhang W, Guhathakurta S, Brown MA, et al Machine learning approaches for estimating commercial building energy consumption. Applied Energy. 2017;208(Supplement C):889–904. 10.1016/j.apenergy.2017.09.060.

[8] Voyant C, Notton G, Kalogirou S, Nivet ML, Paoli C, Motte F, et al Machine learning methods for solar radiation forecasting: A review. Renewable Energy. 2017;105(Supplement C):569–582. 10.1016/j.renene.2016.12.095.

[9] Adya M, Collopy F. How effective are neural networks at forecasting and prediction? A review and evaluation. Journal of Forecasting. 1998;17(56):481–495. 10.1002/(SICI)1099-131X(1998090)17:5/6<481::AID-FOR709>3.0.CO;2-Q

[10] Chatfield C. Neural networks: Forecasting breakthrough or passing fad? International Journal of Forecasting. 1993;9(1):1–3. 10.1016/0169-2070(93)90043-M.

[11] Sharda R, Patil RB. Connectionist approach to time series prediction: An empirical test. Journal of Intelligent Manufacturing. 1992;3(1):317–323. 10.1007/BF01577272

[12] Crone SF, Hibon M, Nikolopoulos K. Advances in forecasting with neural networks? Empirical evidence from the NN3 competition on time series prediction. International Journal of Forecasting. 2011;27(3):635–660. 10.1016/j.ijforecast.2011.04.001.

[13] Keogh E, Kasetty S. On the Need for Time Series Data Mining Benchmarks: A Survey and Empirical Demonstration. Data Mining and Knowledge Discovery. 2003;7(4):349–371. 10.1023/A:1024988512476

[14] Zhang GP. Avoiding Pitfalls in Neural Network Research. IEEE Transactions on Systems, Man, and Cybernetics, Part C (Applications and Reviews). 2007;37 10.1109/TSMCC.2006.876059

[15] Ahmed NK, Atiya AF, Gayar NE, El-Shishiny H. An Empirical Comparison of Machine Learning Models for Time Series Forecasting. Econometric Reviews. 2010;29(5–6):594–621. 10.1080/07474938.2010.481556

[16] Makridakis S, Hibon M. The M3-Competition: results, conclusions and implications. International Journal of Forecasting. 2000;16(4):451–476. 10.1016/S0169-2070(00)00057-1

[17] Goodfellow I, Bengio Y, Courville A. Deep Learning. MIT Press; 2016.

[18] Wang J, Wang J. Forecasting stochastic neural network based on financial empirical mode decomposition. Neural Networks. 2017;90:8–20. 10.1016/j.neunet.2017.03.004. 28364677

[19] Zhao L. Neural Networks In Business Time Series Forecasting: Benefits And Problems. Review of Business Information Systems (RBIS). 2009;13(3):57–62.

[20] Assimakopoulos V, Nikolopoulos K. The theta model: a decomposition approach to forecasting. International Journal of Forecasting. 2000;16(4):521–530. 10.1016/S0169-2070(00)00066-2

[21] Ilies I, Jaeger H, Kosuchinas O, Rincon M, VakÄ?nas V, Vaskevicius N. Stepping forward through echoes of the past: Forecasting with Echo State Networks, Technical Report: Jacobs University Bremen; 2007.

[22] Gardner ES. Exponential smoothing: The state of the art-Part II. International Journal of Forecasting. 2006;22(4):637–666. 10.1016/j.ijforecast.2006.03.005

[23] Hamid SA, Habib A. Financial Forecasting with Neural Networks. Academy of Accounting and Financial Studies Journal. 2014;18(4):37–55.

[24] Qiu M, Song Y. Predicting the Direction of Stock Market Index Movement Using an Optimized Artificial Neural Network Model. PLOS ONE. 2016;11(5):1–11. 10.1371/journal.pone.0155133 PMC4873195 27196055

[25] Kock AB, Teräsvirta T. Forecasting Macroeconomic Variables Using Neural Network Models and Three Automated Model Selection Techniques. Econometric Reviews. 2016;35(8–10):1753–1779. 10.1080/07474938.2015.1035163

[26] Gabor MR, Dorgo LA. Neural Networks Versus Box-Jenkins Method for Turnover Forecasting: a Case Study on the Romanian Organisation. Transformations in Business and Economics. 2017;16(1):187–211.

[27] Marr B. The Top 10 AI And Machine Learning Use Cases Everyone Should Know About, Forbes; 2016 Available from: https://www.forbes.com/sites/bernardmarr/2016/09/30/what-are-the-top-10-use-cases-for-machine-learning-and-ai/#2c292bf894c9.

[28] Zhang GP, Qi M. Neural network forecasting for seasonal and trend time series. European Journal of Operational Research. 2005;160(2):501–514. 10.1016/j.ejor.2003.08.037

[29] Alpaydin E. Machine Learning: Introduction to Machine Learning. The MIT Press; 2004.

[30] Hastie T, Tibshirani R, Friedman J. The elements of statistical learning: Data mining, Inference, and Prediction, Second Edition Springer New York; 2009.

[31] Hyndman RJ, Koehler AB. Another look at measures of forecast accuracy. International Journal of Forecasting. 2006;22(4):679–688. 10.1016/j.ijforecast.2006.03.001

[32] Goodwin P, Lawton R. On the asymmetry of the symmetric MAPE. International Journal of Forecasting. 1999;15(4):405–408. 10.1016/S0169-2070(99)00007-2.

[33] Hansen LK, Salamon P. Neural Network Ensembles. IEEE Transactions on Pattern Analysis and Machine Intelligence. 1990;12(10):993–1001. 10.1109/34.58871

[34] Gardner ES. Exponential smoothing: the state of the art. Journal of Forecasting. 1985;4(1):1–28. 10.1002/for.3980040103

[35] Andrawis RR, Atiya AF, El-Shishiny H. Forecast combinations of computational intelligence and linear models for the NN5 time series forecasting competition. International Journal of Forecasting. 2011;27(3):672–688. 10.1016/j.ijforecast.2010.09.005

[36] Hyndman R, Khandakar Y. Automatic time series forecasting: the forecast package for R. Journal of Statistical Software. 2008;26(3):1–22.19777145

[37] Hyndman RJ, Koehler AB, Snyder RD, Grose S. A state space framework for automatic forecasting using exponential smoothing methods. International Journal of Forecasting. 2002;18(3):439–454. 10.1016/S0169-2070(01)00110-8

[38] Connor JT, Martin RD, Atlas LE. Recurrent neural networks and robust time series prediction. IEEE transactions on neural networks. 1994;5(2):240–54. 10.1109/72.279188 18267794

[39] Makridakis SG, Wheelwright SC, Hyndman RJ. Forecasting: Methods and applications (Third Edition). New York: Wiley; 1998.

[40] Jain LC, Medsker LR. Recurrent Neural Networks: Design and Applications. 1st ed Boca Raton, FL, USA: CRC Press, Inc; 1999.

[41] Hochreiter S, Schmidhuber J. Long Short-Term Memory. Neural Computation. 1997;9(8):1735–1780. 10.1162/neco.1997.9.8.1735 9377276

[42] Lippmann RP. An Introduction to Computing with Neural Nets. IEEE ASSP Magazine. 1987;4(2):4–22. 10.1109/MASSP.1987.1165576

[43] Møller M. A scaled conjugate gradient algorithm for fast supervised learning. Neural networks. 1993;6:525–533. 10.1016/S0893-6080(05)80056-5

[44] Kourentzes N, Barrow DK, Crone SF. Neural network ensemble operators for time series forecasting. Expert Systems with Applications. 2014;41(9):4235–4244. 10.1016/j.eswa.2013.12.011

[45] Bergmeir C, Benitez JM. Neural Networks in R Using the Stuttgart Neural Network Simulator: RSNNS. Journal of Statistical Software. 2012;46(7):1–26. 10.18637/jss.v046.i0722837731

[46] MacKay DJC. Bayesian Interpolation. Neural Computation. 1992;4(3):415–447. 10.1162/neco.1992.4.3.415

[47] Dan Foresee F, Hagan MT. Gauss-Newton approximation to bayesian learning. In: IEEE International Conference on Neural Networks—Conference Proceedings. vol. 3; 1997. p. 1930–1935.

[48] Nguyen D, Widrow B. Improving the learning speed of 2-layer neural networks by choosing initial values of the adaptive weights. IJCNN Int Joint Conf Neural Networks. 1990;13:C21.

[49] Rodriguez PP, Gianola D. brnn: Bayesian Regularization for Feed-Forward Neural Networks; 2016 Available from: https://cran.r-project.org/package=brnn.

[50] Specht DF. A general regression neural network. IEEE Transactions on Neural Networks. 1991;2(6):568–576. 10.1109/72.97934 18282872

[51] Chasset P -O. GRNN: General regression neural network for the statistical software R; 2013 Available from: http://flow.chasset.net/r-grnn/.

[52] Venables WN, Ripley BD. Modern Applied Statistics with S 4th ed New York: Springer; 2002 Available from: http://www.stats.ox.ac.uk/pub/MASS4.

[53] Breiman L. Classification and Regression Trees. Boca Raton, FL: Chapman & Hall; 1993.

[54] Therneau T, Atkinson B, Ripley B. rpart: Recursive Partitioning and Regression Trees; 2015 Available from: https://cran.r-project.org/package=rpart.

[55] Schölkopf B, Smola AJ. Learning with kernel: Support Vector Machines, Regularization, Optimization and Beyond. The MIT Press; 2001 Available from: http://citeseerx.ist.psu.edu/viewdoc/download?doi=10.1.1.167.5140{&}rep=rep1{&}type=pdf.

[56] Meyer D, Dimitriadou E, Hornik K, Weingessel A, Leisch F. e1071: Misc Functions of the Department of Statistics, Probability Theory Group, TU Wien; 2017 Available from: https://cran.r-project.org/package=e1071.

[57] Rasmussen CE, Williams C. Gaussian Processes for Machine Learning. The MIT Press; 2006.

[58] Karatzoglou A, Smola A, Hornik K, Zeileis A. kernlab—An S4 Package for Kernel Methods in R. Journal of Statistical Software. 2004;11(9):1–20. 10.18637/jss.v011.i09

[59] Elman JL. Finding structure in time. Cognitive Science. 1990;14(2):179–211. 10.1207/s15516709cog1402_1

[60] Chollet F, et al Keras; 2015 https://github.com/keras-team/keras.

[61] Abadi M, Agarwal A, Barham P, Brevdo E, Chen Z, Citro C, et al TensorFlow: Large-Scale Machine Learning on Heterogeneous Systems; 2015 Available from: http://tensorflow.org/.

[62] Gorr WL. Research prospective on neural network forecasting. International Journal of Forecasting. 1994;10(1):1–4. 10.1016/0169-2070(94)90044-2

[63] Nelson M, Hill T, Remus B, O’Connor M. Can neural networks applied to time series forecasting learn seasonal patterns: an empirical investigation. System Sciences, 1994 Proceedings of the Twenty-Seventh Hawaii International Conference on. 1994;3:649–655. 10.1109/HICSS.1994.323316

[64] Cottrell M, Girard B, Girard Y, Mangeas M, Muller C. Neural Modeling for Time Series: A Statistical Stepwise Method for Weight Elimination. IEEE Transactions on Neural Networks. 1995;6(6):1355–1364. 10.1109/72.471372 18263428

[65] Nelson CR, Plosser CR. Trends and random walks in macroeconmic time series. Some evidence and implications. Journal of Monetary Economics. 1982;10(2):139–162. 10.1016/0304-3932(82)90012-5

[66] Box GEP, Cox DR. An Analysis of Transformations. Journal of the Royal Statistical Society Series B (Methodological). 1964;26(2):211–252.

[67] Cox DR, Stuart A. Some Quick Sign Tests for Trend in Location and Dispersion. Biometrika. 1955;42(1–2):80–95. 10.2307/2333424

[68] Sakamoto Y, Ishiguro M, Kitagawa G. Akaike Information Criterion Statistics. D. Reidel Publishing Company; 1986.

[69] Zaremba W, Sutskever I, Vinyals O. Recurrent Neural Network Regularization. CoRR. 2014.

[70] Brown RG. Statistical forecasting for inventory control. New York: McGraw-Hill; 1959.

[71] Box G, Jenkins G. Time Series Analysis: Forecasting and Control. San Francisco: Holden-Day; 1970.

[72] Pearl J. Causality: Models, Reasoning, and Inference. New York: Cambridge University Press; 2000.

[73] Bauwens L, Laurent S, Rombouts JVK. Multivariate GARCH models: a survey. Journal of Applied Econometrics. 2006;21(1):79–109. 10.1002/jae.842

[74] Laurent S, Rombouts JVK, Violante F. On the forecasting accuracy of multivariate GARCH models. Journal of Applied Econometrics. 2012;27(6):934–955. 10.1002/jae.1248

[75] Green KC, Armstrong JS. Simple versus complex forecasting: The evidence. Journal of Business Research. 2015;68(8):1678–1685. 10.1016/j.jbusres.2015.03.026.

[76] Makridakis S, Hibon M. Accuracy of Forecasting: An Empirical Investigation. Journal of the Royal Statistical Society Series A (General). 1979;142(2):97–145. 10.2307/2345077

[77] Spiliotis E, Assimakopoulos V. Are M3 Competition data representative of the reality? Working paper. 2018;.

[78] Kang Y, Hyndman RJ, Smith-Miles K. Visualising forecasting algorithm performance using time series instance spaces. International Journal of Forecasting. 2017;33(2):345–358. 10.1016/j.ijforecast.2016.09.004

[79] Petropoulos F, Makridakis S, Assimakopoulos V, Nikolopoulos K. ‘Horses for Courses’ in demand forecasting. European Journal of Operational Research. 2014;237(1):152–163. 10.1016/j.ejor.2014.02.036

[80] Liu W, Wang Z, Liu X, Zeng N, Liu Y, Alsaadi FE. A survey of deep neural network architectures and their applications. Neurocomputing. 2017;234(Supplement C):11–26. 10.1016/j.neucom.2016.12.038.

[81] Zeng N, Zhang H, Song B, Liu W, Li Y, Dobaie AM. Facial expression recognition via learning deep sparse autoencoders. Neurocomputing. 2018;273(Supplement C):643–649. 10.1016/j.neucom.2017.08.043.

