# Supplementary material for: Statistical and Machine Learning forecasting methods: Concerns and ways forward
Source: PLoS One. 2018 Mar 27;13(3):e0194889. doi: 10.1371/journal.pone.0194889 (PMC5870978; doi:10.1371/journal.pone.0194889)
Supplement: S1 Appendix — The accuracy is evaluated per forecasting horizon first according to sMAPE, and then to MASE. (PDF) [file pone.0194889.s001.pdf]

Table A1. The sMAPE for each of the 18 forecasting horizon and their overall average.

Model	1	2	3	4	5	6	7	8	9	10	11	12	13	14	15	16	17	18	Mean
MLP Iterative	7.98	8.62	9.47	9.81	10.75	10.56	12.07	11.52	12.03	12.86	12.35	13.23	13.44	13.65	14.49	15.53	15.71	17.20	12.29
MLP Direct	8.92	9.88	10.82	11.07	11.82	11.83	13.18	12.81	13.28	14.10	13.59	14.37	14.51	14.93	16.06	16.73	16.76	18.21	13.49
MLP Multi	7.87	8.33	9.39	9.77	10.88	10.92	12.30	11.65	12.25	13.34	12.86	13.77	14.02	14.52	16.01	16.69	16.83	18.38	12.77
BNN Iterative	7.92	8.37	9.46	9.67	10.54	10.39	11.86	11.28	11.73	12.53	12.04	13.03	13.28	13.54	14.33	15.28	15.55	16.82	12.09
BNN Multi	7.95	8.19	9.34	9.75	10.74	10.90	12.36	11.63	12.38	13.20	12.86	13.79	13.93	14.64	15.77	16.46	16.68	18.25	12.71
Naïve2	10.75	9.07	11.50	11.92	10.38	11.07	11.31	12.59	13.24	12.28	12.98	12.38	12.89	14.02	15.87	16.01	15.18	16.49	12.77
SES	8.57	7.58	9.43	9.83	9.62	9.98	10.54	10.34	11.24	10.80	10.87	11.29	11.29	12.78	14.12	14.92	14.06	15.46	11.26
Holt	7.97	7.75	8.92	9.51	10.16	10.08	11.11	10.45	11.33	11.67	11.02	11.53	12.34	12.72	14.14	15.21	15.21	16.12	11.51
Damped	8.14	7.44	9.00	9.52	9.72	9.97	10.60	10.10	10.83	10.72	10.62	10.93	11.27	12.25	13.67	14.46	13.93	15.19	11.02
Comb	8.18	7.50	9.00	9.50	9.70	9.84	10.54	10.00	10.87	10.75	10.47	10.82	11.18	12.15	13.53	14.51	13.96	14.99	10.97
Theta	8.20	7.59	9.02	9.44	9.68	9.82	10.54	9.98	10.73	10.77	10.25	10.91	11.06	12.04	13.21	14.14	13.81	14.86	10.89
ARIMA	7.78	7.56	9.00	9.31	9.84	10.11	10.88	10.62	11.27	11.40	10.88	11.41	11.85	12.38	13.71	14.34	14.72	16.03	11.28
ETS	8.06	7.57	9.17	9.60	9.94	10.08	10.91	10.66	11.09	11.21	10.82	11.16	11.80	12.37	13.83	14.65	14.35	15.45	11.26

Table A2. The MASE for each of the 18 forecasting horizon and their overall average.

Model	1	2	3	4	5	6	7	8	9	10	11	12	13	14	15	16	17	18	Mean
MLP Iterative	0.51	0.54	0.61	0.72	0.76	0.81	0.90	0.91	0.97	1.05	1.02	1.05	1.10	1.14	1.20	1.29	1.28	1.43	0.96
MLP Direct	0.61	0.65	0.72	0.82	0.85	0.91	1.01	1.03	1.08	1.18	1.14	1.18	1.23	1.29	1.35	1.42	1.42	1.56	1.08
MLP Multi	0.50	0.53	0.61	0.71	0.76	0.81	0.93	0.91	0.98	1.11	1.07	1.12	1.18	1.24	1.33	1.42	1.43	1.62	1.01
BNN Iterative	0.50	0.53	0.60	0.70	0.73	0.78	0.87	0.88	0.93	1.00	0.97	1.01	1.06	1.09	1.15	1.24	1.30	1.37	0.93
BNN Multi	0.50	0.52	0.60	0.71	0.75	0.81	0.91	0.93	1.00	1.09	1.08	1.11	1.19	1.26	1.32	1.40	1.41	1.55	1.01
Naïve2	0.66	0.59	0.72	0.87	0.82	0.91	0.96	1.03	1.07	1.11	1.08	1.04	1.16	1.19	1.36	1.45	1.42	1.55	1.05
SES	0.53	0.51	0.62	0.76	0.76	0.84	0.90	0.92	0.96	1.02	0.96	1.01	1.08	1.13	1.27	1.39	1.37	1.50	0.97
Holt	0.51	0.50	0.58	0.72	0.74	0.80	0.86	0.88	0.92	1.00	0.92	0.95	1.06	1.07	1.21	1.33	1.34	1.47	0.94
Damped	0.51	0.49	0.59	0.73	0.73	0.81	0.86	0.87	0.90	0.97	0.91	0.93	1.02	1.04	1.18	1.30	1.30	1.42	0.92
Comb	0.51	0.49	0.59	0.72	0.73	0.80	0.85	0.86	0.90	0.96	0.89	0.92	1.01	1.03	1.17	1.29	1.28	1.40	0.91
Theta	0.51	0.50	0.59	0.72	0.72	0.80	0.85	0.85	0.88	0.95	0.87	0.91	0.99	1.02	1.14	1.25	1.24	1.36	0.90
ARIMA	0.48	0.48	0.56	0.68	0.70	0.78	0.84	0.85	0.90	0.96	0.89	0.92	0.99	1.03	1.14	1.23	1.25	1.40	0.89
ETS	0.51	0.49	0.60	0.73	0.74	0.81	0.87	0.90	0.92	0.98	0.91	0.95	1.03	1.04	1.19	1.29	1.30	1.42	0.92
